# Deacetylation of YAP1 Promotes the Resistance to Chemo- and Targeted Therapy in FLT3-ITD^+^ AML Cells

**DOI:** 10.3389/fcell.2022.842214

**Published:** 2022-05-17

**Authors:** Panpan Feng, Jingru Zhang, Juan Zhang, Xiaomin Liu, Lina Pan, Dawei Chen, Min Ji, Fei Lu, Peng Li, Guosheng Li, Tao Sun, Jingxin Li, Jingjing Ye, Chunyan Ji

**Affiliations:** ^1^ Department of Hematology, Qilu Hospital, Cheeloo College of Medicine, Shandong University, Jinan, China; ^2^ Laboratory of Medical Chemistry, GIGA-Stem Cells, Faculty of Medicine, University of Liege, CHU, Liege, Belgium; ^3^ Department of Physiology, School of Basic Medical Sciences, Cheeloo College of Medicine, Shandong University, Jinan, China

**Keywords:** FLT3-ITD^+^ AML, resistance, Yap1, HDAC10, PARP1

## Abstract

The FLT3-ITD mutation occurs in about 30% of acute myeloid leukemia (AML) and is associated with poor prognosis. However, FLT3 inhibitors are only partially effective and prone to acquired resistance. Here, we identified Yes-associated protein 1 (YAP1) as a tumor suppressor in FLT3-ITD^+^ AML. YAP1 inactivation conferred FLT3-ITD^+^ AML cell resistance to chemo- and targeted therapy. Mass spectrometric assay revealed that DNA damage repair gene poly (ADP-ribose) polymerase 1 (PARP1) might be the downstream of YAP1, and the pro-proliferative effect by YAP1 knockdown was partly reversed *via* PARP1 inhibitor. Importantly, histone deacetylase 10 (HDAC10) contributed to decreased YAP1 acetylation levels through histone H3 lysine 27 (H3K27) acetylation, leading to the reduced nuclear accumulation of YAP1. Selective HDAC10 inhibitor chidamide or HDAC10 knockdown activated YAP1, enhanced DNA damage, and significantly attenuated FLT3-ITD^+^ AML cell resistance. In addition, combination chidamide with FLT3 inhibitors or chemotherapy agents synergistically inhibited growth and increased apoptosis of FLT3-ITD^+^ AML cell lines and acquired resistant cells from the relapse FLT3-ITD^+^ AML patients. These findings demonstrate that the HDAC10-YAP1-PARP1 axis maintains FLT3-ITD^+^ AML cells and targeting this axis might improve clinical outcomes in FLT3-ITD^+^ AML patients.

## Introduction

Acute myeloid leukemia (AML) represents a group of highly heterogeneous myeloid malignancies characterized by acquired genetic abnormalities in hematopoietic progenitors. Among these recurrent mutations, internal tandem duplications (ITDs) of the FMS-like receptor tyrosine kinase 3 (FLT3) are the most frequent, presenting in 25–30% AML patients. Patients with FLT3-ITD mutation have a high risk of relapse and low cure rates (Kiyoi et al., 2005; De Kouchkovsky et al., 2016; Daver et al., 2019). Despite several FLT3 inhibitors, such as sorafenib, midostaurin, gilteritinib, and quizartinib (AC220), having been approved for targeted therapy in clinical usage, a large number of FLT3-ITD^+^ AML cases only achieve a transient clinical response and gradually become resistant to monotherapy or in combination with conventional chemotherapy (Papaemmanuil et al., 2016; Daver et al., 2019). It is now widely accepted that the defects in the core machinery of the DNA damage pathway contribute to drug resistance (Caracciolo et al., 2021; Wang et al., 2021). Moreover, we have previously shown the important function of DNA damage repair gene poly (ADP-ribose) polymerase 1 (PARP1) in the progression of AML (Wang et al., 2015). Thus, exploration of novel effective therapeutic agents is urgently needed to prevent FLT3-ITD^+^ AML resistance and improve patient prognosis. Identifying molecular mechanisms involved in FLT3-ITD^+^ AML maintenance and drug resistance may therefore be the key to providing such novel treatment approaches.

Yes-associated protein 1 (YAP1) is one of the key transcriptional effectors in the Hippo pathway, which is a crucial regulator of tissue homeostasis, organ size, and cell proliferation. Once cells proliferate to a high density and the Hippo pathway is activated (Zhao et al., 2007), phosphorylated YAP1 will promote its cytoplasmic retention and subsequent degradation by the ubiquitin proteasome pathway (Yu and Guan, 2013; Ma et al., 2016; Meng et al., 2016). Relatively, unphosphorylated YAP1 entering into the nucleus, where YAP1 activates transcription factors, alters the expression of genes involved in cell proliferation and apoptosis (Pobbati and Hong, 2013). YAP1 has been demonstrated to play a critical role in the development and progression of cancers (Pan, 2010; Moroishi et al., 2015). YAP1 has recently been defined to limit cancer transformation in response to DNA damage (Pefani and O'Neill, 2016). It behaves as an oncogene in several solid tumors and hematologic malignances, including chronic myeloid leukemia (CML) (Bertini et al., 2009; Cottini et al., 2014; Li et al., 2016). Nevertheless, YAP1 was also found to be a tumor suppressor gene. Specifically, restoring the level of YAP1 significantly inhibits proliferation and increases chemosensitivity of hepatic carcinoma cells (Bai et al., 2013). Therefore, the role of YAP1 is cell context–dependent and its effect on FLT3-ITD^+^ AML maintenance needs to be further clarified.

Aberrant histone deacetylase (HDAC) expression and the association with poor prognosis are well reported in several types of human solid cancers and specifically in AML (West and Johnstone, 2014; San José-Enériz et al., 2019; Zhou et al., 2021). Histone modifications have been central in the understanding of post-translational modifications and their effects on the regulation of gene expression. Therefore, it is not surprising that aberrant activity of HDACs can cause deregulated gene expression and protein function, which may serve as pivotal mechanisms to promote tumorigenesis and drug resistance. In the case of AML, mutations in HDAC genes have not been detected, but interestingly, it has been described that oncogenic fusion proteins can recruit HDACs to specific gene promoters to drive leukemogenesis. For instance, AML1-ETO chimeric fusion protein recruits HDAC1, HDAC2, and HDAC3, silencing AML1 target genes and therefore leading to differentiation arrest and transformation (Liu et al., 2006). Moreover, HDAC3 enhances the DNA damage repair capability of leukemia cells through activating AKT and thereby protecting leukemia cells from chemotoxicity, suggesting that HDACs can participate in drug resistance by facilitating DNA damage repair (Lahue and Frizzell, 2012). However, no reports have been published regarding the role of HDACs in the regulation of YAP1 in leukemia. Our preliminary study demonstrated that HDAC inhibitor chidamide treatment concomitantly facilitated the nuclear localization of YAP1 and enhanced its acetylation level in FLT3-ITD^+^ AML cells, which led us to conduct the current study to determine whether and how dysregulation of YAP1 could contribute to chidamide-induced inhibitory effects in human AML.

In this study, our data identified a suppressive role of YAP1 in FLT3-ITD^+^ AML. These results provided supportive evidence that YAP1 inactivation could be involved in the resistance to chemo- and targeted therapy in FLT3-ITD^+^ AML cells. Selective HDAC10 inhibitor in combination with FLT3 inhibitors or chemotherapy showed a synergistic cytotoxic effect on the resistant FLT3-ITD^+^ AML patient cells. We proposed that simultaneous targeting of FLT3 and HDAC10 may be a promising strategy to improve the treatment outcome of FLT3-ITD^+^ AML.

## Materials and Methods

### Patient Samples

Bone marrow (BM) samples from FLT3-ITD^+^ AML patients were obtained from Qilu Hospital, Shandong University, including cases with sorafenib-sensitive FLT3-ITD^+^ AML (*n* = 5) and cases with relapsed/refractory FLT3-ITD^+^ AML (*n* = 5). FLT3-ITD^+^ AML cases were defined according to the classification in the NCCN guidelines and received FLT3 inhibitor sorafenib combined with chemotherapy. The detailed information of AML patients is provided in Supplementary Table S1. The study is approved by the Qilu Hospital Ethics Review Board in accordance with the Declaration of Helsinki. Acquisition of bone marrow samples was performed with the informed consent of the patients. Mononuclear cells were isolated by density gradient centrifugation using Lymphocyte Separation Medium (Haoyang, China) and cultured in IMDM (Gibco, United States) supplemented with 10% fetal bovine serum (Gibco, United States), and 1% penicillin/streptomycin.

### Cell Lines and Culture

The FLT3-ITD^+^ human leukemic cell lines MV4-11 cell line and MOLM13 were grown at 37°C, 5% CO_2_ in Iscove’s Modified Dulbecco’s Medium (IMDM, Gibco, United States) supplemented with 10% fetal bovine serum (FBS, Gibco, United States) and 1% penicillin/streptomycin, respectively.

Sorafenib-resistant cell models are developed in the laboratory by long-time exposure of cells growing in cell culture to incremental concentrations of sorafenib. Briefly, the method comprises two steps. In the adaptation stage, MV4-11 cells and MOLM13 were treated with sorafenib for 48 h by a stepwise increase in drug concentration from 10 to 50 nmol/L (10, 25, and 50 nmol/L). After each dose-induced step, we discarded apoptotic cells and amplified surviving cells in a sorafenib-free culture medium. The cells were then exposed to the next increment of sorafenib. In the consolidation stage, previously selected cells were treated with different final concentrations of sorafenib (50 nmol/L) until they grew normally in a conditioned medium. The surviving daughter resistant cells are then compared to the parental sensitive cells using combination cell viability assays, CCK8 assay.

### Reagents

Chidamide (CS055, purity >95%) was supplied by Chipscreen Bioscience Ltd. (Shenzhen, China) and dissolved in dimethyl sulfoxide (DMSO) to obtain a stock solution of 10 mM. PARP1 inhibitor Olaparib (AZD2281, KU0059436) was supplied by Selleck (United States) and dissolved in DMSO to obtain a stock solution of 10 mM. Sorafenib, Midostaurin, and AC220 were supplied by Medchemexpress (United States) and dissolved in DMSO to obtain a stock solution of 10 mM. The stock solutions were stored at −80 °C. They were diluted to the required concentrations in subsequent experiments with culture medium.

### Lentiviral Transduction

Lentiviral constructs expressing YAP1 were purchased from Genechem (Shanghai, China). We introduced object lentivirus or control lentivirus into MV4-11 cells seeded in 24-well plate for 5 × 10^4^ cells. After 3 days, the infected cells were subject to puromycin (4 μg/ml) selection for 7 days. Puromycin-resistant colonies were picked up and then expanded in 6-well plates.

### siRNA Transfection

YAP1 siRNA was purchased from Genepharma (Shanghai, China). MV4-11 cells were transfected with 100 nM siRNA using lipofectamineTM 2000 (Invitrogen). Then, 24 h post transfection, the transfected cells were treated with 10 μM of Olaparib and viable cell counts were quantified by CCK8 assay, and 48 h post transfection, the transfected cells were collected for Western blot.

### Total RNA Isolation and RT-qPCR

Total RNA was isolated using Trizol (TaKaRa, China) according to the manufacturer’s protocols. RNA (1 μg) was reverse transcribed into cDNA using RT reagent kit (TaKaRa, China). The quantitative real-time polymerase chain reactions were performed by an Applied Biosystem 7900HT System (ABI) with appropriate primers, using SYBR Green PCR Master Mix (Toyobo, Japan). The human housekeeping gene GAPDH was used as the RNA-loading control. Each sample was amplified in a 10 μl reaction volume according to the manufacturer’s instructions. The primers used in this assay were: YAP1, forward primer: CCA​CAG​GCA​ATG​CGG​AAT​ATC, reverse primer: GGT​GCC​ACT​GTT​AAG​GAA​AGG; VCP, forward primer: AGT​TGC​CAA​GGA​TGT​GGA​CT, reverse primer: GGT​TTG​TCT​GCC​TCT​CTC​GT; PARP1, forward primer: GAA​TGC​CAG​CGT​TAC​AAG​CC, reverse primer: TCT​CCC​TGA​GAC​GTA​TGG​CA; GAPDH, forward primer: GCA​CCG​TCA​AGG​CTG​AGA​AC, reverse primer: TGG​TGA​AGA​CGC​CAG​TGG​A.

### Western Blot

The protein expression levels were determined by staining with primary antibodies and relevant secondary (1:5000, Zhongshanjinqiao, China) antibodies. The primary antibodies [PARP, #9532; γ-H2AX, #9718; TATA-binding protein (TBP), #44059; H3, #4499; AceH3(K27), #8173, cell signaling technology, Herts, United Kingdom; YAP1, #13584-1-AP, Proteintech, China] were diluted at 1:1000 in 5% fat-free milk phosphate-buffered saline (PBS, Servicebio, China) with 0.05% TWEEN 20 (Solarbio, China) (PBST). Anti-β-actin (1:1000, #ZM-0003SMA, Zhongshanjinqiao, China) was used as the loading control of cell lines, and GAPDH (1:1000, #60004-1-Ig, Proteintech, Wuhan, China) was used as the loading control of primary cells from the relapsed FLT3-ITD^+^ AML patients. The protein bands were visualized using a FluorChem E Chemiluminescent Western Blot Imaging System (Cell Biosciences). The bidimensional optical densities of proteins on the films were quantified and analyzed with Quantity One software (ImageJ), and the relative values were calculated by comparing with the loading control.

### Cell Counting Kit-8

The cytotoxic effects of homoharringtonine calculated by comparing with ringtonine (HHT), cytarabine (Ara-C), sorafenib, midostaruin, and AC220 with or without chidamide on MV4-11 cells were determined by the Cell Counting Kit-8 (CCK-8, Beibo, China) assay. Cells (1 × 10^5^ cells/well) were seeded in 96-well plates containing 100 μl growth medium and treated with designated doses of HHT, Ara-C, sorafenib, midostaruin, and AC220 in combination with or without 2, 4 μM chidamide and incubated at 37°C in a 5% CO_2_ incubator for 48 h; CCK-8 reagents (10 μl/well) were then added and continued to incubate for an additional 2–4 h; finally, absorbance (450 nm) was measured by a microplate reader (Thermo Scientific) and IC50 determination was calculated.

### Apoptosis

Cells were treated with HHT, Ara-C, sorafenib, midostaruin, and AC220 with or without chidamide for 24 h, stained with Annexin V/PI (BestBio, Shanghai, China) or Annexin V/7-AAD (BestBio, Shanghai, China) for 15 min at room temperature in the dark according to the manufacturer’s instructions, and apoptosis was analyzed by flow cytometry (Beckman Coulter).

### Subcellular Fractionation

The chidamide-treated MV4-11 cells were lysed with a Nuclear Protein Extraction Kit (BestBio, Shanghai, China) in proper conditions to obtain two fractions: one fraction enriched in cytoplasm/membrane proteins, and one in nuclear proteins. The cells were treated with 200 μl buffer A added with proteases and phosphatase inhibitors. After 15 min of mild agitation in ice, the cells were centrifuged at 400×g at 4°C. The supernatant was then centrifuged at 15000 × g to clarify the extract and the supernatant, representing the cytoplasm/membrane enriched fraction, was collected. The pellet obtained from the 400 × g centrifugation was treated with 100 μl buffer B added with protease and phosphatase inhibitors, mildly agitated for 40 min in ice, and then centrifuged at 15000 × g at 4C. The supernatant was collected and the nuclear enriched fraction was represented.

### Immunofluorescence

Cells were cultured with or without 2, 4 μM chidamide for 24 h. The cells were harvested and dropped in glass coverslips, which were then fixed with 4% paraformaldehyde for 20 min, followed by washing thrice with PBS, permeabilizing with 0.1% Triton X-100 (Sigma) for 15 min, and blocking with 5% BSA in PBS for 1 h at room temperature (RT). The samples were then stained overnight at 4°C with primary antibody against YAP1 (1:100, Cell Signaling, United Kingdom), followed by incubation with FITC goat anti-rabbit IgG (Sigma) for 1 h at RT in the dark, and then were counterstained using DAPI. Subsequently, the coverslips were mounted on glass slides and cell nuclei. The cells were scanned and images were captured by a fluorescence microscope.

### RNA Sequencing

We performed RNA sequencing of MV4-11 cells cultured in the presence of low-dose sorafenib of 10 nM for 24 days. The total RNAs were isolated from Trizol. Complementary DNA (cDNA) libraries were prepared with the RNA Hyper Prep Kit according to the manufacturer’s instructions, and high-throughput sequencing of the cDNA libraries was performed on an Illumina X ten platform for 150 bp paired-end sequencing. |Log2Fold change| >1 and false discovery rate *p* < 0.05 were set as the cutoffs to screen for differentially expressed genes. The fragments per kilobase of transcript per million reads mapped (FPKM) assay was applied to measure transcript expression levels in RNA sequencing isoform quantification software.

### Co-Immunoprecipitation

MV4-11 cells were treated with or without 2 μM chidamide for 24 h. Cell lysates were prepared by lysing cells in buffer containing 25 mM Tris-HCl (pH 7.4), 150 mM NaCl, 1% NP-40, 1 mM EDTA, and protease and phosphatase inhibitors (Thermo Scientific, #87787), and cell debris was removed by centrifugation. Total protein was incubated with anti-YAP1 or anti IgG antibodies (16 h, 4°C, rotation), and then protein A/G PLUS Agarose beads were added (20 μl, 1 h, 4°C, rotation; Santa Cruz Biotechnology). The captured agarose beads Ab–Ag complexes were washed (five times, PBS) and detected by Western blot with rabbit polyclonal YAP1 antibody (#13584-1-AP, Proteintech, Wuhan) and pan-acetylation antibody (#66289-1-Ig, Proteintech, Wuhan).

### Immunoprecipitation and Mass Spectrometry

Cell lysates were prepared by lysing cells in buffer containing 25 mM Tris-HCl (pH 7.4), 150 mM NaCl, 1% NP-40, 1 mM EDTA, and protease and phosphatase inhibitors (#87787, Thermo Scientific). Total protein was then incubated with anti-YAP1 or anti IgG antibodies (16 h, 4°C, rotation), and protein A/G PLUS Agarose beads were added (20 μl, 1 h, 4°C, rotation). The captured agarose beads Ab–Ag complexes were washed (five times, PBS) and detected by sodium dodecyl sulfate polyacrylamide gel electrophoresis (SDS-page), followed by dyeing the strips with coomassie brilliant blue, cutting the strips in the lanes, and mixing them for mass spectrometry analysis, which was performed by Thermo’s Q Exactive™ LC/MS system. Next, the YAP1-interacting proteins of control and YO groups selected Score Sequest HT ≥ 1.5, deleted contaminating proteins, and removed high molecular weight proteins and negative reference proteins of the IgG group.

### Statistical Analyses

Data are presented as mean ± standard deviation (SD). Each experiment was repeated in triplicate at least. Differences between two groups were analyzed using an unpaired Student t-test. Differences between three or four groups were analyzed using one-way ANOVA or two-way ANOVA followed by LSD test with normally distributed data. The Mann–Whitney U test was used for cases with unequal variances. *p* < 0.05 was considered statistically significant.

## Result

### YAP1 Played a Tumor Suppressor Role in FLT3-ITD^+^ AML

**FIGURE 1:**
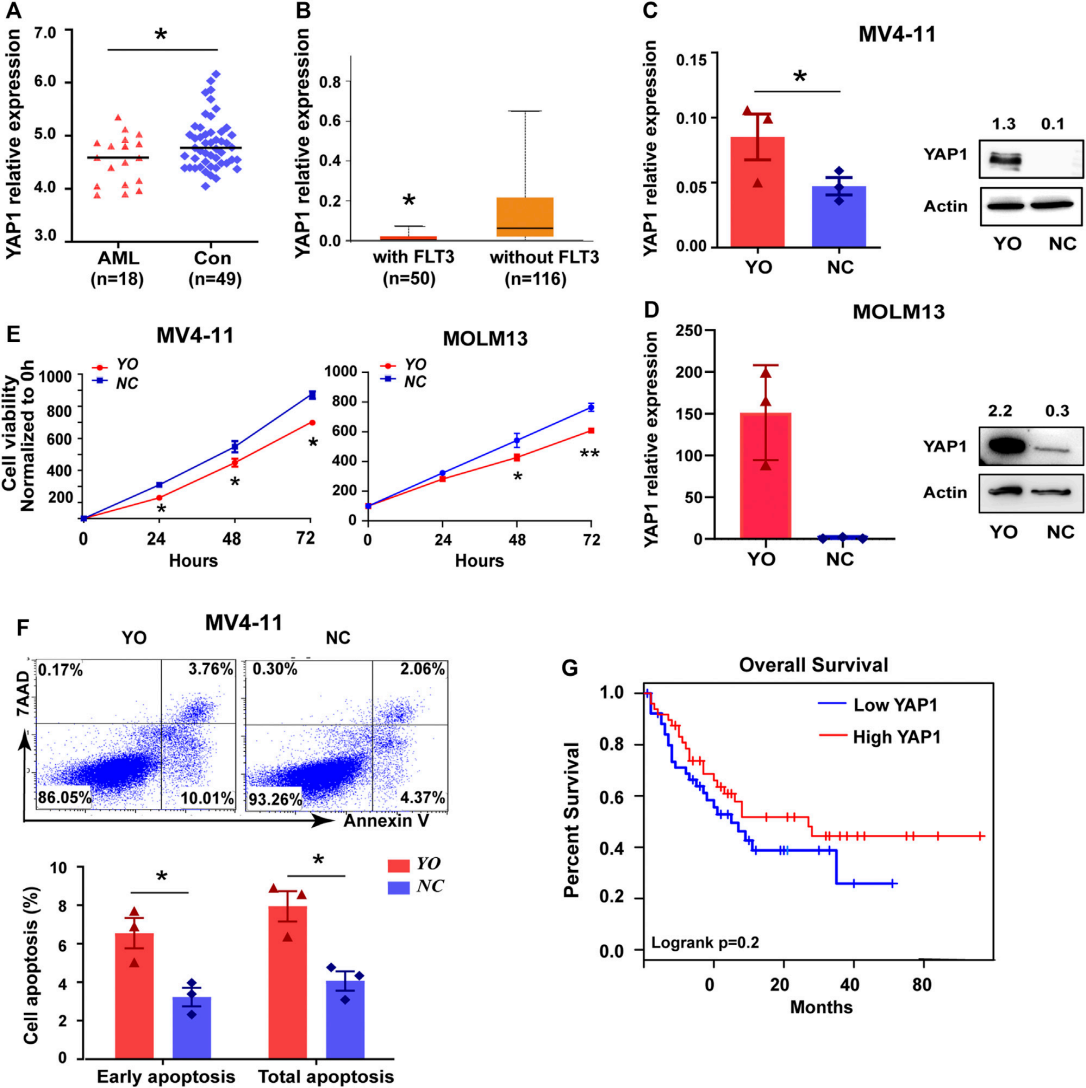
YAP1 plays a tumor suppressor role in FLT3-ITD^+^ AML. **(A)** YAP1 expression in AML patient samples (*n* = 18, red) and control BM samples (*n* = 49, blue) from GEO database, GSE48558. **(B)** YAP1 expression in FLT3 mutant AML patients (*n* = 50, red) and without FLT3 mutant AML patients (*n* = 116, orange) from TCGA database. **(C)** and **(D)** YAP1 overexpression (YO) or control (NC) MV4-11 cells and MOLM13 cells were selected by puromycin, followed by RT-qPCR and Western blot with indicated antibodies. **(E)** Proliferation of YO and NC cells were assessed by CCK8 assays, and proliferation rates at 0, 12, 24, 48, and 72 h were calculated normalized to the absorbance at 0 h. **(F)** Apoptosis of YO and NC cells were measured by flow cytometry. **(G)** OS analysis of AML patients (*n* = 101) using the expression of YAP1 from TCGA database. Data are shown as mean ± SD of three independent experiments; *p < 0.05.

It is well known that YAP1 plays a pivotal role in the pathogenesis and progression of cancers, including leukemia (Li et al., 2016). Due to the uncertain effect of YAP1 on FLT3-ITD^+^ AML, we performed the bioinformatics analysis of YAP1. Based on GEO and TCGA database, the expression level of YAP1 was lower in AML cells than that in normal cells (Figure 1A and Supplementary Figure S1A). Compared to FLT3-ITD^WT^ AML patients, the YAP1 expression was even lower in FLT3-ITD^+^ AML patients (Figure 1B). Similar results were found in FLT3-ITD^WT^ and FLT3-ITD^+^ cell lines (Supplementary Figure S1B,C). To explore the potential role of YAP1, we overexpressed YAP1 in MV4-11 cells and MOLM13 cells by lentivirus infection. Overexpression of YAP1 was verified by RT-qPCR and Western blot (Figures 1C,D). YAP1 upregulation markedly inhibited growth and induced apoptosis of FLT3-ITD^+^ AML cells (Figures 1E,F). In addition, the decreased expression of YAP1 was associated with worse prognosis in AML patients (Figure 1G). These results demonstrated that YAP1 might be a tumor suppressor in FLT3-ITD^+^ AML.

### Inactivation of YAP1 Conferred FLT3-ITD^+^ AML Cell Resistance to Chemo- and Targeted Therapy

**FIGURE 2:**
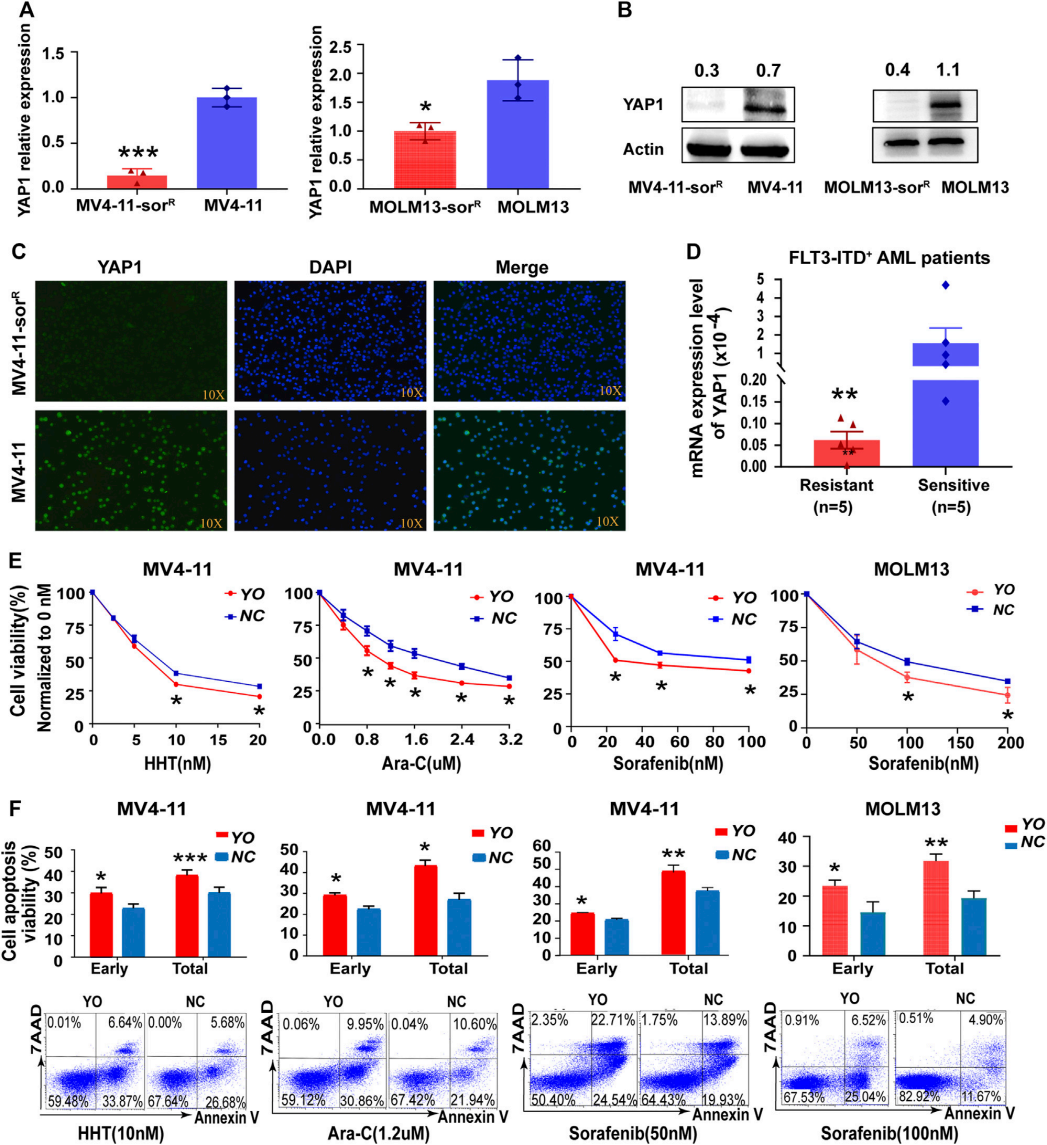
Inactivation of YAP1 promotes drug resistance to chemo- and targeted therapy in FLT3-ITD^+^ AML cells. **(A–C)** YAP1 expression was measured by RT-qPCR, Western blot, and immunofluorescence in sorafenib-resistant cells and the parental cells of MV4-11 and MOLM13. **(D)** YAP1 expression was analyzed in resistant FLT3-ITD^+^ AML patients (*n* = 5, red) and sensitive FLT3-ITD^+^ AML patients (*n* = 5, blue). **(E)** Cell growth inhibition of YO and NC cells was calculated after 48 h of treatment with serial dilutions of homoharringtonine (HHT), cytarabine (Ara-C), and sorafenib. **(F)** YO and NC cells were treated with HHT (10 nM), Ara-C (1.2 μM), and sorafenib (50 nM or 100 nM) for 24 h to measure apoptosis by flow cytometry. Data are shown as mean ± SD; *p < 0.05; **p < 0.01; ***p < 0.001.

To further identify whether YAP1 was involved in the drug resistance of FLT3-ITD^+^ AML, we established two new sorafenib-resistant cell lines, MV4-11-Sor^R^ and MOLM13-Sor^R^. The resistance index was calculated according to the IC50 of resistant cells relative to the parental cells (5-fold) (Supplementary Figure S2A). RNA sequencing offered 285 altered KEGG pathways that conferred resistance to sorafenib, and the Hippo signaling pathway was identified among these pathways (Supplementary Figure S2B,C). Interestingly, the resistant cells showed an even lower YAP1 level than the parental cells (Figures 2A–C). We also found that the YAP1 mRNA level was lower in resistant FLT3-ITD^+^ AML samples (Figure 2D). YAP1 upregulation decreased the IC50 of FLT3 inhibitor sorafenib, HHT, and cytarabine Ara-C, confirming that YAP1 activation could resensitize FLT3-ITD^+^ AML cells (Figure 2E). In addition, apoptotic cell death induced by FLT3 inhibitor and chemotherapeutics increased after YAP1 overexpression in MV4-11 cells (Figure 2F). Our data indicated that downregulation of YAP1 was sufficient to confer resistance to chemo- and targeted therapy in FLT3-ITD^+^ AML.

### YAP1 Suppression Promoted Resistance Through DNA Damage Response Dependent of PARP1 in FLT3-ITD^+^ AML Cells

**FIGURE 3:**
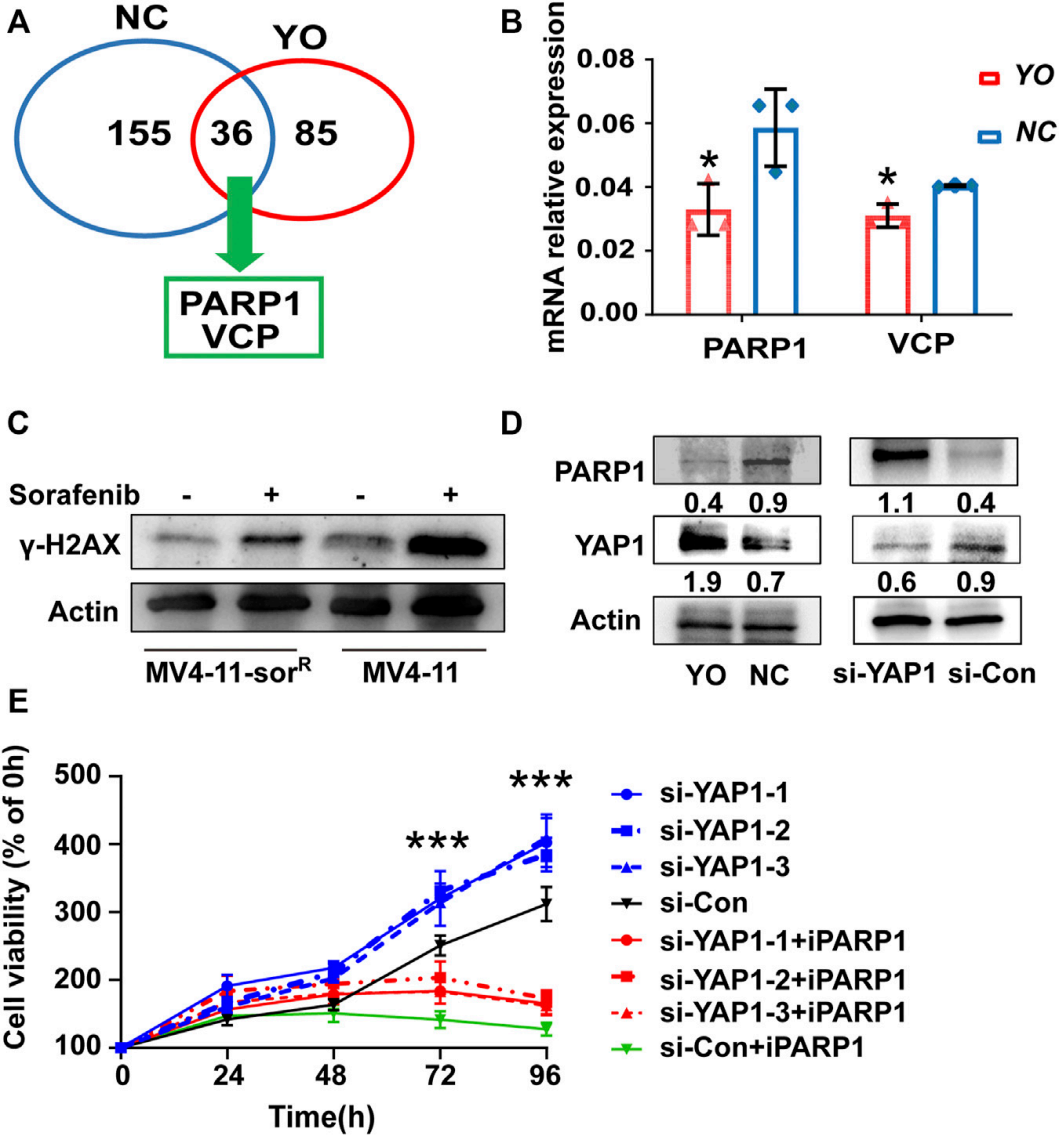
YAP1 suppression promoted FLT3-ITD^+^ AML cell resistance through DNA damage response dependent of PARP1. **(A)** Mass spectrometry analyzed selected proteins (Score Sequest HT ≥ 1.5) of YO (red) and NC (blue) cells. **(B)** RT-qPCR detected the mRNA expression level of PARP1 and VCP in YO and NC cells. **(C)** γ-H2AX protein expression of MV4-11-sor^R^ and MV4-11 cells was examined after 6 h of treatment with or without sorafenib (50 nM). **(D)** PARP1 and YAP1 protein expression was analyzed by Western blot in YO and NC cells as well as in YAP1 knockdown (si-YAP1) and non-silencing scrambled control (si-Con) cells, respectively. **(E)** Proliferation of si-YAP1 and si-Con cells with or without PARP1 inhibitor Olaparib (iPARP1) was assessed by CCK8 assays, and viable cell rates at 12, 24, 48, 72, and 96 h were calculated normalized to the absorbance at 0 h. Data are shown as mean ± SD of three independent experiments; *p < 0.05; **p < 0.01; ***p < 0.001.

Subsequently, we conducted mass spectrometric analysis to explore the molecular mechanism of YAP1 that confers acquired resistance in FLT3-ITD^+^ AML cells. Data showed that a total of 155 proteins were screened out by YAP1 antibody pull-down in control cells; meanwhile, 85 proteins were screened out in YAP1-overexpressed MV4-11-Sor^R^ cells (Supplementary Material). Interestingly, YAP1 upregulation resulted in an obvious change of 36 overlap proteins, including DNA damage repair gene PARP1 and valosin-containing protein (VCP) (Figure 3A). Consistently, RT-qPCR analysis revealed that the PARP1 transcript level reduced by 35%, following activation of YAP1 (Figure 3B). Next, we investigated the DNA damage degree in response to sorafenib. The Western blot analysis of γ-H2AX showed that MV4-11 cells had a distinct higher level of DNA damage response after sorafenib treatment than MV4-11-Sor^R^ cells, demonstrating that MV4-11-Sor^R^ cells are less than MV4-11 cells in the level of damaged DNA induced by sorafenib (Figure 3C).

To further examine whether PARP1 was the downstream target of YAP1, we carried out gain- and loss-of-function experiments. The siRNA-mediated knockdown of YAP1 was able to increase the protein level of PARP1, and YAP1 activation had an opposite effect (Figure 3D). Notably, the CCK8 proliferation assay showed that small molecular PARP1 inhibitor Olaparib alleviated the pro-proliferative function of YAP1 knockdown to a certain degree (Figure 3E). These results provided the mechanistic evidence that YAP1-mediated FLT3-ITD^+^ AML resistance was in part due to PARP1 activation involved in DNA damage repair.

### HDAC10 Contributed to the Resistance of FLT3-ITD^+^ AML Cells and Selective HDAC10 Inhibitor Chidamide Synergistically Enhanced the Cytotoxic Effect of FLT3 Inhibitors

**FIGURE 4:**
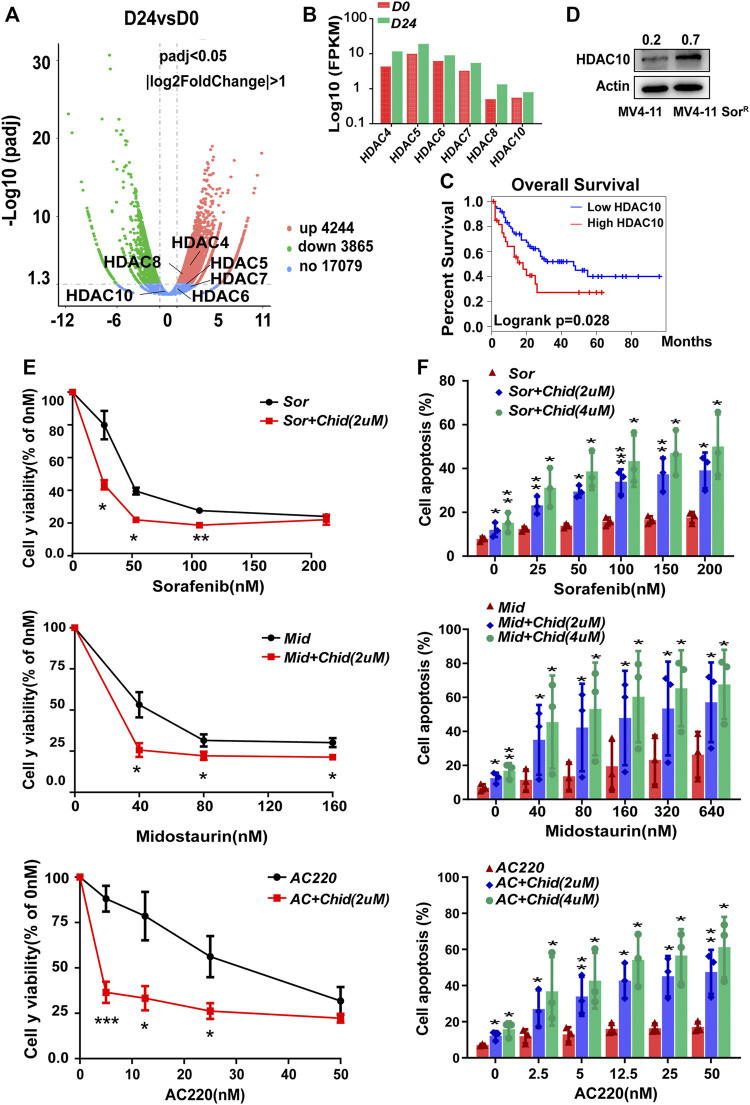
HDAC10 contributed to the resistance of FLT3-ITD^+^ AML cells, and selective HDAC10 inhibitor chidamide synergistically enhanced the cytotoxic effect of FLT3 inhibitors. **(A)** Volcano plot showed both positively (red) and negatively (green) selected genes by the RNA sequence at 24 d post sorafenib treatment. **(B)** Validation of selected genes in the Volcano plot using Fpkm assay in RNA sequence. **(C)** OS analysis of AML patients (*n* = 144) using the expression of HDAC10 from TCGA database. **(D)** Expression level of HDAC10 was analyzed by Western blot in MV4-11 cells and MV4-11-sor^R^ cells. **(E)** Cell growth inhibition of MV4-11 cells was calculated after 48 h of treatment with serial dilutions of FLT3 inhibitors sorafenib, midostaurin, and AC220 combined with chidamide (2 mM). **(F)** MV4-11 cells were treated with serial dilutions of sorafenib, midostaurin, and AC220 combined with chidamide (2uM, 4uM) for 24 h to measure apoptosis by flow cytometry. Data are shown as mean ± SD of three independent experiments; *p < 0.05; **p < 0.01; ***p < 0.001.

Although HDAC family members were known as mediators of AML chemoresistance, there was little knowledge about their effects on FLT3-ITD^+^ AML cell resistance (San José-Enériz et al., 2019). In the first instance, we sought to identify altered HDACs that confer acquired resistance of FLT3-ITD^+^ AML cells, following 24 days of post-sorafenib treatment. The volcano plot showed both upregulated and downregulated selected genes as assessed by RNA sequencing in MV4-11-Sor^R^ cells (Figure 4A). By FPKM assay, the HDAC4-8 and HDAC10 mRNA expression levels were increased, which might confer resistance (Figure 4B). We subsequently analyzed those upregulated selected genes through TCGA database; only the high level of HDAC10 was indicative of poor survival in AML patients (Figure 4C and Supplementary Figure S3). The HDAC10 protein level was also elevated in MV4-11-SorR cells (Figure 4D). Therefore, HDAC10 might facilitate the resistance of MV4-11 cells. In addition, selective HDAC10 inhibitor chidamide combined with FLT3 inhibitors had a higher inhibitory rate in MV4-11 cells than that in FLT3 inhibitors alone, suggesting that HDAC10 suppression could enhance the sensitivity of FLT3 inhibitors, including sorafenib, midostaurin, and AC220 (Figure 4E). The apoptosis analysis also demonstrated a similar result (Figure 4F). Therefore, HDAC10 might play an important role in poor prognosis and resistance of FLT3-ITD^+^ AML.

### Acetylation Level of YAP1 was Enhanced by HDAC10 Knockdown Through H3K27 Acetylation, Accompanied With the Increased Nuclear Accumulation of YAP1 and the Decreased Expression of PARP1

**FIGURE 5:**
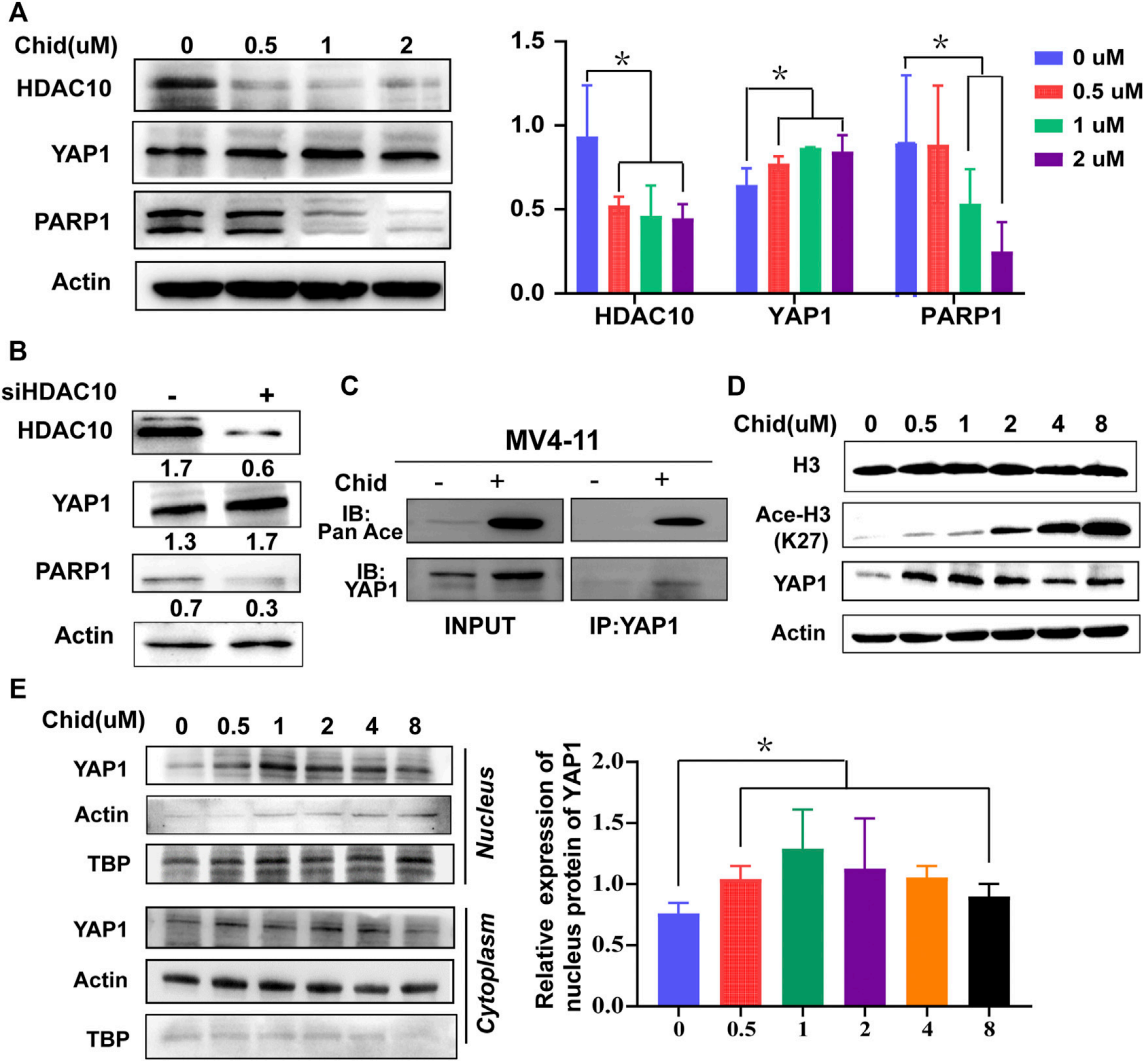
Acetylation level of YAP1 was enhanced by HDAC10 knockdown through H3K27 acetylation, accompanied with the increased nuclear accumulation of YAP1 and the decreased expression of PARP1. **(A)** Expression levels of HDAC10, YAP1, and PARP1 were analyzed by Western blot in MV4-11 cells after treatment with chidamide (0, 0.5, 1, 2*μ*M) for 24 h. **(B)** Expression levels of HDAC10, YAP1, and PARP1 were analyzed by Western blot in YAP1 knockdown (si-YAP1) and non-silencing scrambled control (si-Con) MV4-11 cells. **(C)** Acetylation level of YAP1 was assessed by co-immunoprecipitation assay in chidamide-treated MV4-11 cells. **(D)** Expression levels of Histone3, acetyl H3K27, and YAP1 were analyzed by Western blot in MV4-11 cells after treatment with chidamide (0, 0.5, 1, 2, 4, 8uM) for 24 h. **(E)** Western blot analyzed the cytoplasm and nuclear levels of YAP1.

Next, we investigated whether YAP1 was deacetylated by HDAC10 and further identified the specific lysine acetylation site. HDAC10 expression was dose-dependently decreased by chidamide treatment, leading to the increased expression of YAP1 and the decreased expression of its downstream target PARP1 (Figure 5A). Furthermore, HDAC10 knockdown by siRNA has consistent results with chidamide (Figure 5B). Through the enrichment of Ac-lysine, we uncovered that the YAP1 acetylation level markedly boosted following chidamide treatment (Figure 5C). Western blot revealed that the acetylated level of histone H3 lysine 27 (H3K27) was markedly increased after chidamide treatment (Figure 5D), suggesting that YAP1 might exert its transcriptional control through H3K27 acetylation. In addition, chidamide could induce the nuclear accumulation of YAP1 in a dose-dependent manner as demonstrated by the Western blot analysis (Figure 5E). Thus, these data indicate that inhibition of HDAC10 by chidamide could enhance the acetylation of YAP1 through acetylated H3K27 and impair the ability of nuclear YAP1-induced DNA damage repair.

### Chidamide Potentiated YAP1 Expression and the Combination of Chidamide and FLT3 Inhibitors or Chemotherapy Agents Synergistically Promoted Apoptosis in the Acquired Resistant AML Cells *ex vivo*

**FIGURE 6:**
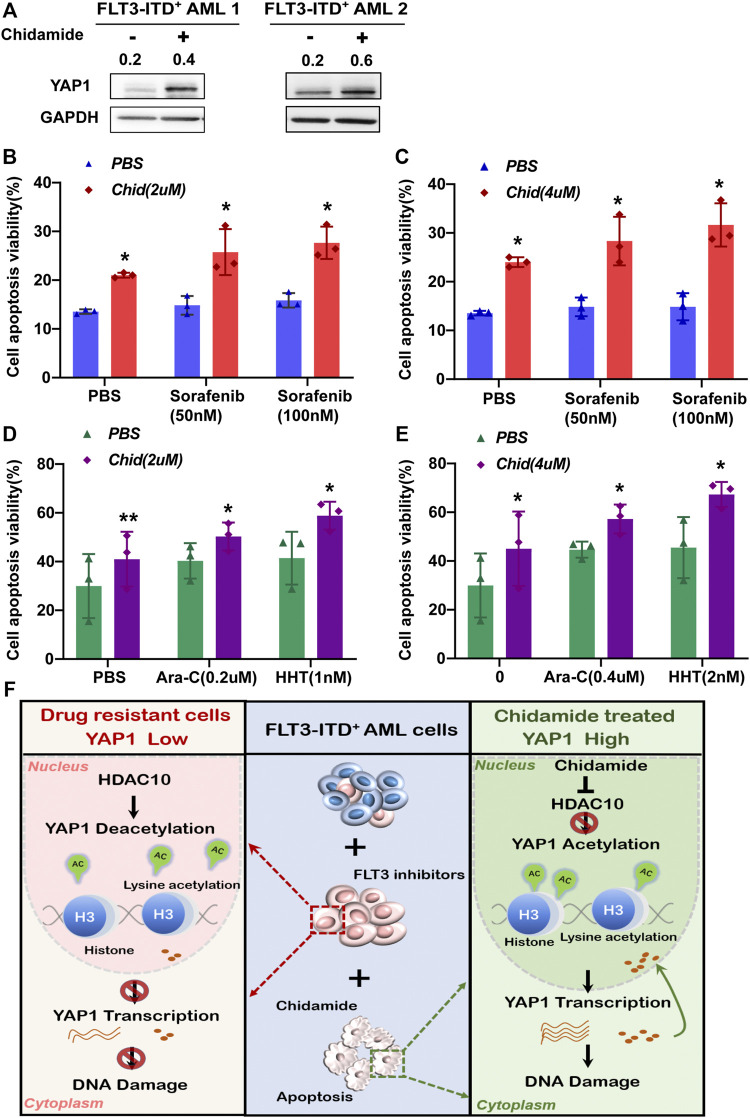
Chidamide-potentiated YAP1 expression and the combination chidamide with FLT3 inhibitors or chemotherapy agents synergistically promoted apoptosis in the acquired resistant FLT3-ITD^+^ AML cells ***ex vivo.* (A)** Expression level of YAP1 was analyzed by Western blot in FLT3-ITD^+^ AML cells after treatment with chidamide for 24 h. **(B)** and **(C)** Apoptosis rate was analyzed by flow cytometry in relapse FLT3-ITD^+^ AML patient cells after treatment with chidamide (2 or 4*μ*M) combined with sorafenib (50 or 100 nM). **(D)** and **(E)** Apoptosis rate was analyzed by flow cytometry in relapse FLT3-ITD^+^ AML patient cells after treatment with chidamide (2 or 4nM) combined with HHT (1 or 2 nM) or Ara-C (0.2 or 0.4*μ*M). **(F)** Schematic summary of this study. HDAC10 mediates the chemoresistance of FLT3-ITD^+^ AML cells to FLT3 inhibitors by deacetylating YAP1. Targeting the HDAC10 sensitizes FLT3-ITD^+^ AML cells to FLT3 inhibitor treatment by enhancing acetylation of YAP1 through acetyl H3K27, promoting its transcriptional control, and strengthening nuclear YAP1-induced DNA damage repair. Data are shown as mean ± SD of three independent experiments; *p < 0.05; **p < 0.01.

To further evaluate the survival-inhibitory effects of either chidamide and/or FLT3 inhibitors or chemotherapy agents on the acquired resistant AML cells, we performed an apoptosis assay in the sorted BMMCs from the relapsed FLT3-ITD^+^ AML patients previously administrated with sorafenib plus HHT and Ara-C. Those acquired resistant AML cells were regarded as insensitive to sorafenib, HHT, and Ara-C. The Western blot analysis showed that a lower YAP1 level was detected in acquired resistant cells and chidamide was able to increase the YAP1 protein levels (Figure 6A). As shown in Figure 6B, we found that 2 or 4 mm of chidamide together with sorafenib exhibited a more pro-apoptosis effect in acquired resistant FLT3-ITD^+^ AML cells. Furthermore, the combination of chidamide and HHT or Ara-C synergistically decreased the amount of viable resistant AML cells (Figure 6C). Together, these results demonstrated that chidamide potentiated YAP1 expression, selectively inhibited cell survival, and the combination of chidamide and FLT3 inhibitors or chemotherapy agents displayed more lethality in acquired resistant FLT3-ITD^+^ AML cells *ex vivo*.

## Discussion

Despite the recent advances in AML therapy, the 5-year survival rate of FLT3-ITD^+^ AML patients remains very low (De Kouchkovsky et al., 2016; Daver et al., 2019). In such patients, FLT3 inhibitors are limited by transient clinical responses and the development of acquired resistance that are assumed to be the source of treatment failure. Therefore, a better understanding of molecular events contributing to drug resistance would aid in the development of strategies to improve treatment outcomes of FLT3-ITD^+^ AML patients. In the current study, we characterized the role for YAP1, a new tumor suppressor, in the resistance to chemo- and targeted therapy of FLT3-ITD^+^ AML cells through DNA damage repair. We also demonstrated that HDAC10 downregulation could increase the acetylated level and nuclear accumulation of YAP1, and selective HDAC10 inhibitor in combination with FLT3 inhibitors or chemotherapy showed a synergistic cytotoxic effect on the resistant FLT3-ITD^+^ AML patient cells.

We reported that the expression level of YAP1 was lower in AML cells than that in normal cells, which was even lower in FLT3-ITD^+^ AML patients than in FLT3-ITD^WT^ AML patients. These data for the first time implicated that YAP1 might play a critical role in FLT3-ITD^+^ AML. YAP1, an essential component of the Hippo pathway, is identified as an oncoprotein which participated in the progression of various malignancies (Kowalik et al., 2011; Avruch et al., 2012). Moreover, targeting YAP1 might enhance the response of CML cells to timatinib *via* downregulating the expression of c-MYC and surviving (Li et al., 2016). However, our results suggested YAP1 to be a negative regulator of drug resistance in FLT3-ITD^+^ AML. We believe that the divergent results are not contradictory. Since the gene-regulated network is abundant, they are not only regulated by signals of proliferation and anti-apoptosis (Yu et al., 2010) but also have a relationship with epigenetic regulation, such as methylation and acetylation. Consequently, there may be other pathways that exist which can regulate YAP1 without the Hippo signaling pathway. Here, we clarified that YAP1-mediated FLT3-ITD^+^ AML resistance was in part due to PARP1 activation involved in DNA damage repair. Accordingly, YAP1 is also shown to enhance DNA damage–induced apoptosis of hepatocellular carcinoma cells by modulation of p53 (Bai et al., 2013).

The various functions of YAP1 were determined by abundant transcription factors, which could bind to YAP1 (Wang et al., 2009). In our study, HDAC10 downregulation was found to promote YAP1 acetylation through H3K27 and thus induce nuclear accumulation of YAP1. Consistently, recent data showed that YAP1 largely exerts its transcriptional control by acetylation through HDAC family members, including sirtuin-1 (SIRT1) and HDAC1 (Hata et al., 2012; Demchak et al., 2014; Yuan et al., 2019). SIRT1 is reported to participate in the YAP acetylation of specific residue (K494), and SIRT1-mediated deacetylation is responsible for the regulatory network of YAP and p53, which may contribute to lung tumorigenesis (Yuan et al., 2019). Moreover, the SIRT1-YAP signaling pathway in which YAP is regulated by SIRT1-mediated deacetylation promotes gastric cancer cell growth through an endocrine mechanism (Li et al., 2017). In addition, SIRT1 is activated by the c-MYC oncogenic network in human FLT3-ITD^+^ AML leukemia stem cells (LSCs), which contributes to their maintenance and drug resistance. Inhibition of this oncogenic network could be an attractive approach for targeting FLT3-ITD^+^ AML LSCs to improve treatment outcomes (Li et al., 2014). Recent data also demonstrate that HDAC8 upregulation is an important mechanism to resist FLT3 inhibitors and promote leukemia maintenance; thereafter, HDAC8 inhibition in combination with FLT3 inhibitor treatment could be a promising strategy to treat FLT3-ITD^+^ AML (Long et al., 2020). Together, we speculate that YAP1 deacetylated by HDAC10 upregulation might be a possible mechanism of drug resistance, and a combination of HDAC10 inhibitors and FLT3 inhibitors or chemotherapy would be a promising strategy to overcome drug resistance and achieve sustained remission in FLT3-ITD^+^ AML.

HDAC inhibitors are considered to possess therapeutic properties in various types of cancers, including lung cancer, prostate cancer, breast cancer, multiple myeloma, and lymphoma (Duvic et al., 2007; Crisanti et al., 2009; Huang et al., 2009; Ogawa et al., 2016). It has currently emerged as a promising therapeutic strategy for cancer therapy. Indeed, HDAC inhibitors have shown an ability to induce differentiation and apoptosis in AML (San José-Enériz et al., 2019; Zhang et al., 2021), leading to a good alternative for treatment, especially for those AML patients not suitable for intensive chemotherapy (Lübbert et al., 2020). Here, we reported that chidamide, a selective HDAC10 inhibitor, combined with FLT3 inhibitors or traditional chemotherapy drugs showed a synergic effect on apoptosis enhancement in the acquired resistant FLT3-ITD^+^ AML cells. Our data provided an alternative combination therapeutic strategy in the treatment of relapsed/refractory FLT3-ITD^+^ AML.

In conclusion, our study improves our understanding of mechanisms underlying the function of YAP1 as an oncogene versus tumor suppressor gene in specific genetic contexts and identifies a novel HDAC10/YAP1/PARP1 axis that mediates drug resistance of FLT3-ITD^+^ AML. Our work provides a basis for advanced testing of YAP1 deacetylation by chidamide *in vivo*.

## Data Availability

The datasets presented in this study can be found in online repositories. The names of the repository/repositories and accession number(s) can be found below: https://www.ncbi.nlm.nih.gov/geo/query/acc.cgi?acc=GSE196258.
