# Supplementary material for: Deacetylation of YAP1 Promotes the Resistance to Chemo- and Targeted Therapy in FLT3-ITD+ AML Cells
Source: Front Cell Dev Biol. 2022 May 17;10:842214. doi: 10.3389/fcell.2022.842214 (PMC9152322; doi:10.3389/fcell.2022.842214)
Supplement: Supplementary file 2 [file Table1.DOCX]

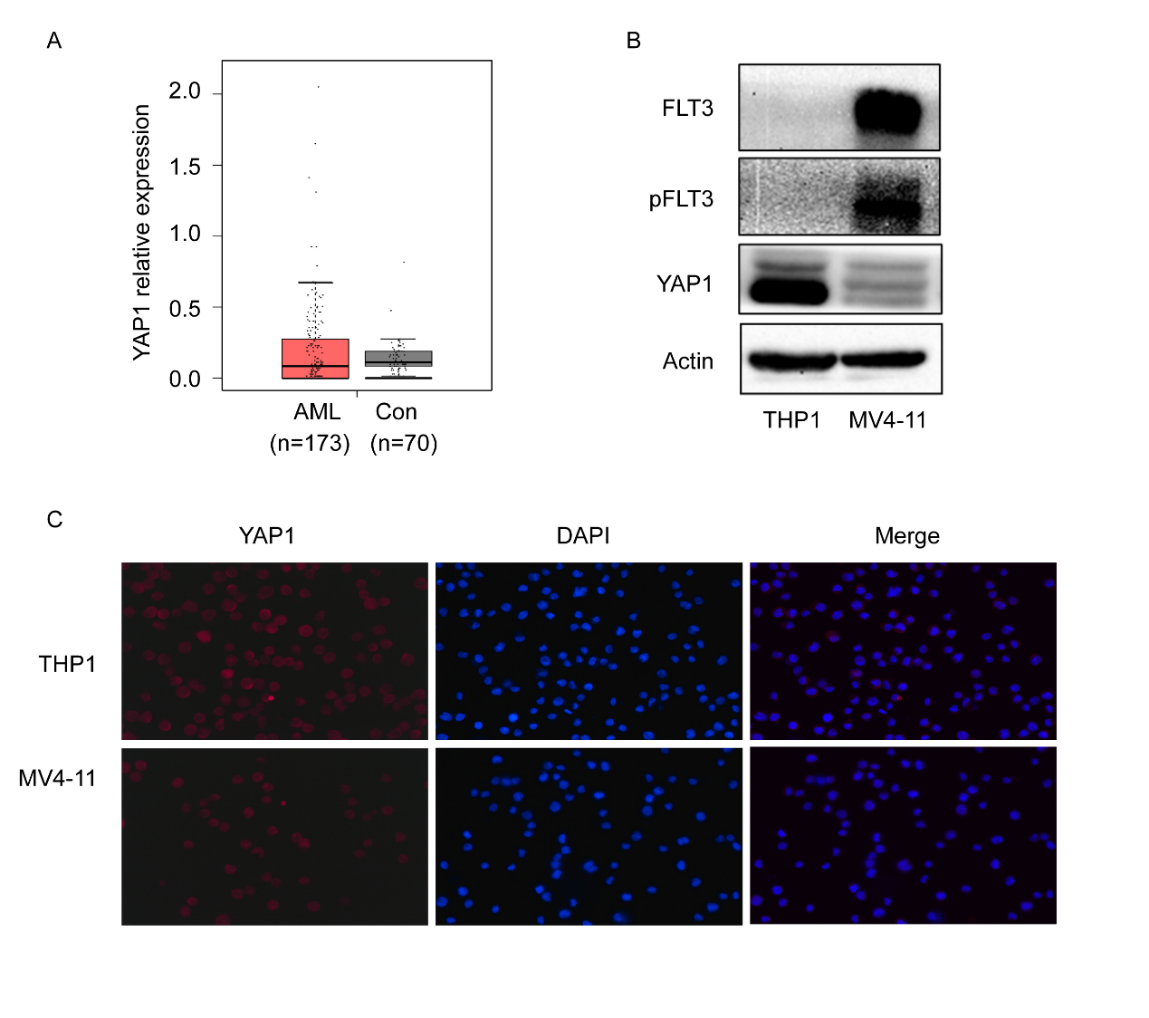


**Supplementary Fig. 1** **Expression of YAP1 in FLT3-ITD^WT^ THP1 and FLT3-ITD^+^ MV4-11 cells. A** YAP1 expression in AML patient samples (n = 173, red) and control BM samples (n = 70, grey) from TCGA database. **B** western blot & **C** Immunofluorescence analyzed the protein level of YAP1 in THP1 cells and MV4-11 cells.


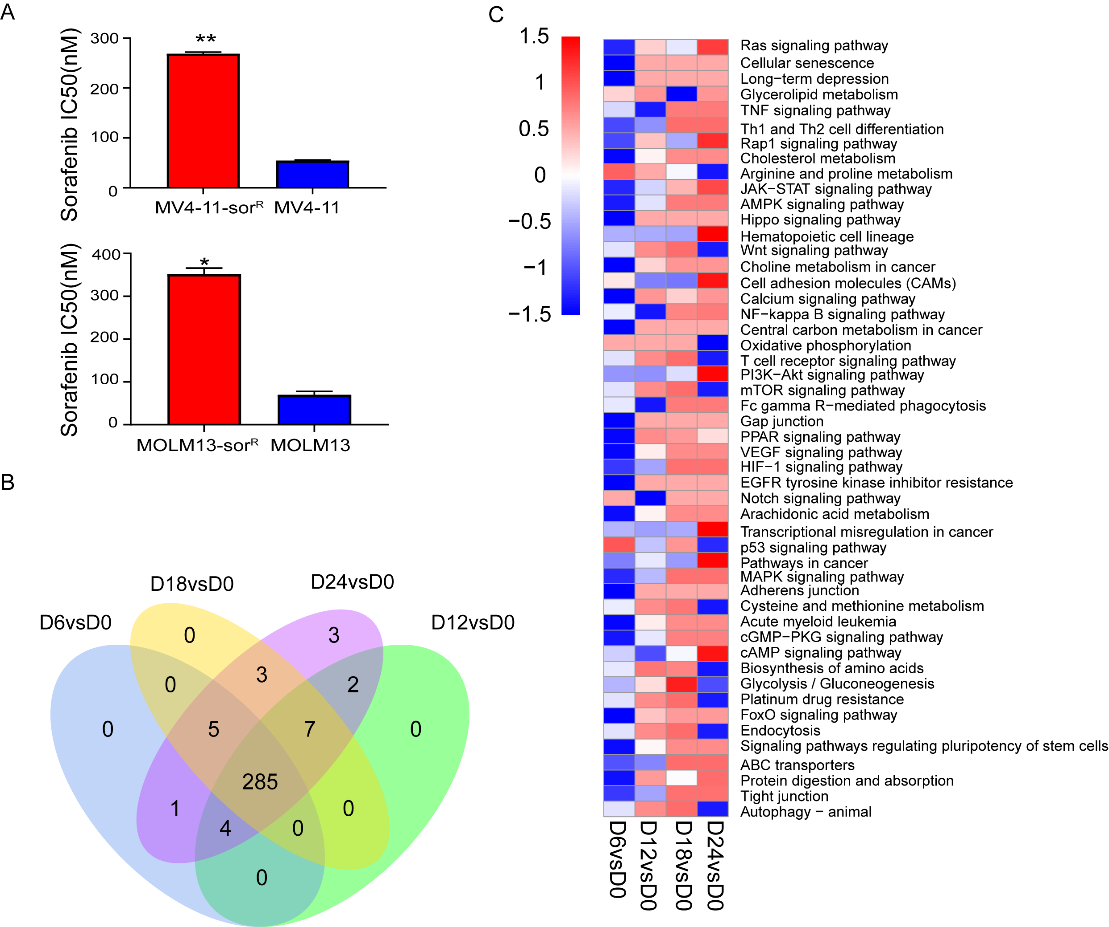


**Supplementary Fig. 2 Hippo/YAP1 is closely associated to the drug sensitivity of FLT3-ITD^+^AML cells. A** IC50 values of MV4-11-Sor^R^ cells and MV4-11 cells were calculated according to cell growth inhibition after 48 h treatment with serial dilutions of Sorafenib. **B**The schematic outline of overlap KEGG pathway. MV4-11 cells were cultured with sorafenib (10nM) for 24 days. RNA sequence sorted out functional categories of altered KEGG pathways at day 6, day 12, day 18 and day 24 post-drug treatment compared to day 0. **C** Heatmap diagram showed the top fifty identiﬁed pathways of the overlap KEGG pathway.


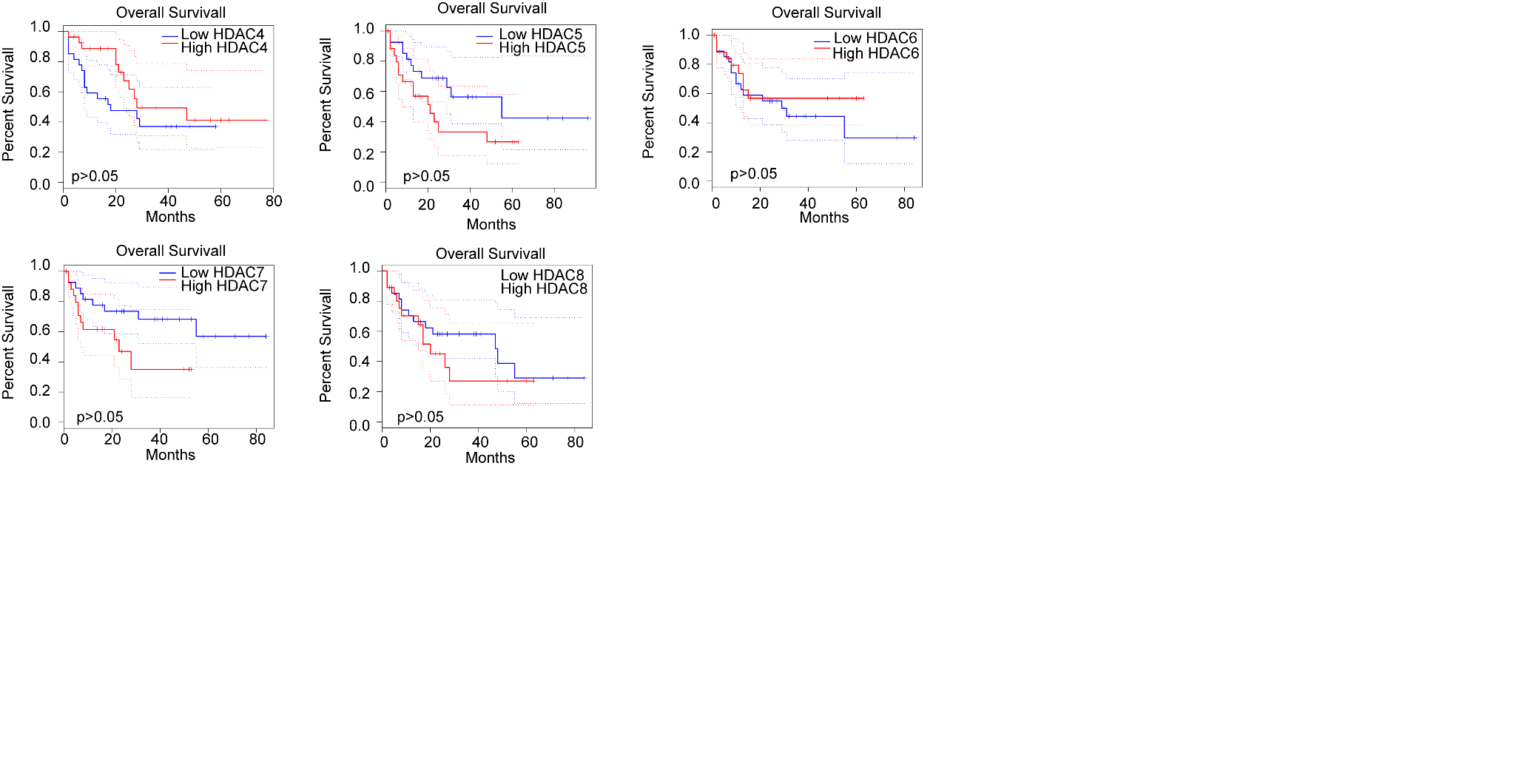


**Supplementary Fig. 3 OS analysis of AML patients using the expression of HDAC4, 5, 6, 7, 8 from TCGA database.**

**Supplementary Table 1: Clinical characteristics of FLT3-ITD^+^ AML patients**

| **Number** | **Age** | **Sex** | **FAB classification** | **WBC** | **HGB** | **PLT** | **Blast in bone**  **marrow (%)** | **FLT3-ITD**  **mutation (%)** | **Therapeutic Response** |
| --- | --- | --- | --- | --- | --- | --- | --- | --- | --- |
| 1 | 74 | Female | M5 | 43.55 | 70 | 44 | 63.6 | 67.47 | Resistant |
| 2 | 44 | Female | M5 | 149.28 | 47 | 28 | 76.0 | 13.34 | Resistant |
| 3 | 64 | Female | M5 | 80.35 | 64 | 46 | 90.0 | 11.54 | Resistant |
| 4 | 19 | Male | M5 | 26.80 | 60 | 42 | 95.0 | 18.51 | Resistant |
| 5 | 53 | Male | M5 | 30.86 | 62 | 12 | 64.0 | 65.39 | Resistant |
| 6 | 50 | Male | M5 | 4.61 | 130 | 69 | 59.0 | 15.00 | Sensitive |
| 7 | 29 | Female | M5 | 45.07 | 74 | 23 | 84.5 | 45.00 | Sensitive |
| 8 | 20 | Male | M5 | 186.72 | 108 | 39 | 85.0 | 11.07 | Sensitive |
| 9 | 30 | Female | M4b | 55.53 | 102 | 74 | 70.0 | 22.50 | Sensitive |
| 10 | 30 | Male | M5 | 54.24 | 113 | 75 | 86.0 | 46.59 | Sensitive |
